# Generation of Inducible CRISPRi and CRISPRa Human Stromal/Stem Cell Lines for Controlled Target Gene Transcription during Lineage Differentiation

**DOI:** 10.1155/2020/8857344

**Published:** 2020-08-19

**Authors:** Li Chen, Kaikai Shi, Weimin Qiu, Lars Aagaard, Moustapha Kassem

**Affiliations:** ^1^Department of Endocrinology and Metabolism, Molecular Endocrinology Laboratory (KMEB), Odense University Hospital, University of Southern Denmark, Denmark; ^2^Department of Pathology and Physiopathology, Guilin Medical University, Guilin, 541004 Guangxi, China; ^3^Department of Biomedicine, Aarhus University, Aarhus C, Denmark; ^4^Department of Cellular and Molecular Medicine, Danish Stem Cell Center (DanStem), University of Copenhagen, 2200 Copenhagen, Denmark

## Abstract

**Background:**

Human bone marrow stromal/stem cells (hMSCs, also known as the skeletal stem cells or mesenchymal stem cells) are being employed to study lineage fate determination to osteoblasts, adipocytes, and chondrocytes. However, mechanistic studies employing hMSC have been hampered by the difficulty of deriving genetically modified cell lines due to the low and unstable transfection efficiency.

**Methods:**

We infected hMSC with a CRISPR/Cas9 lentivirus system, with specific inducible dCas9-coupled transcription activator or repressor: dCas9-KRAB or dCas9-VP64, respectively, and established two hMSC lines (hMSC-CRISPRi and hMSC-CRISPRa) that can inhibit or activate gene expression, respectively. The two cell lines showed similar cell morphology, cell growth kinetics, and similar lineage differentiation potentials as the parental hMSC line. The expression of KRAB-dCas9 or VP64-dCas9 was controlled by the presence or absence of doxycycline (Dox) in the cell culturing medium. To demonstrate the functionality of the dCas9-effector hMSC system, we tested controlled expression of alkaline phosphatase (ALP) gene through transfection with the same single ALP sgRNA.

**Results:**

In the presence of Dox, the expression of ALP showed 60-90% inhibition in hMSC-CRISPRi while ALP showed more than 20-fold increased expression in hMSC-CRISPRa. As expected, the ALP was functionally active and the cells showed evidence for inhibition or enhancement of in vitro osteoblast differentiation, respectively.

**Conclusion:**

hMSC-CRISPRi and hMSC-CRISPRa are useful resources to study genes and genetic pathways regulating lineage-specific differentiation of hMSC.

## 1. Background

Human bone marrow stromal/stem cells (hMSCs, also known as human skeletal or mesenchymal stem cells) are clonal cells present within the bone marrow stroma and are capable of differentiation into various mesoderm-type lineage cells, e.g., osteoblasts, adipocytes and chondrocytes [1]. hMSC has been extensively employed to study the molecular mechanisms of lineage commitment and differentiation and to identify novel factors regulating differentiation processes [2]. We have previously employed global methods of proteomics and transcriptomics to identify specific factors and signaling pathways controlling hMSC differentiation [3–6]. However, follow-up studies focusing on specific factors or a signaling pathway have been hampered by the difficulty in achieving stable hMSC lines that either are deficient or overexpress the target genes of interest at a specific time point of differentiation stages. Moreover, conventional viral mediated gene overexpression in hMSC is technically expensive and time-consuming.

Type II CRISPR-Cas9 system (Clustered Regularly Interspaced Palindromic Repeats–CRISPR-associated 9) is a novel and powerful technology to manipulate gene expression. It is developed from the bacterial immune system for cleaving foreign DNA [7] and is composed of Cas9 endonuclease and a target-identifying CRISPR RNA (single guide RNA, sgRNA). The sgRNA targets a 18-25 base pair sequence of target gene and thus guides Cas9 to specific DNA sites where it creates a blunt-ended double-stranded break (DSB) within the sequence by its endonuclease activity [8, 9]. This DSB induces the generation of mutations that may cause a frameshift in gene coding sequence [10, 11]. Alternatively, it can supply a repair template with homology to the cut site and facilitates targeted integration of mutation or insertion [12].

Besides the direct gene code editing, the CRISPR-Cas9 system can be employed for studying genetic and epigenetic regulation. The Cas9-sgRNA complex can act as a scaffold to recruit different transcription effectors to specific DNA sequences, allowing gene transcription regulation with either transcriptional activation (CRISPRa) or repression (CRISPRi). This function requires disruption of Cas9 nuclease activity by introducing mutations into two nuclease domains (the RuvC and HNH domains) of Cas9 resulting in a deactivated-Cas9 (dCas9) [13–15]. Artificial transcription factors (effector) fused with dCas9 to form the dCas9-effector and then paired with specific sgRNA can be used to target different genes. Different effector proteins, such as transcription activators or repressors fused to dCas9, can differently activate (CRISPRa) or interfere (CRISPRi) in gene expression. In addition, the CRISPR-dCas9 system can be coupled with inducible systems allowing dynamic control of gene transcription [16].

In the present study, we examined the possibility of developing universal hMSC lines to be employed for studies of specific gene activation or inhibition. We employed a system where dCas9 is fused with two different transcription effectors for either activation or inhibition of gene transcription. One effector is the VP64 activator, an engineered tetramer of the herpes simplex VP16 transcriptional activator domain, which can activate silent genes or upregulate active genes in mammalian cells [16–19]. The second effector is a transcriptional repressor KRAB (Krüppel-associated box) domain of Kox1, an efficient repressor of gene transcription [20]. By infecting hMSC with dCas9-VP64 or dCas9-KRAB lentiviral vectors, respectively, we selected and obtained two hMSC lines to be employed for an easy and quick approach for the activation or inhibition of targeted genes by transfecting targeted sgRNA, and we also showed that the regulation of gene expression is inducible by addition or removal of doxycycline (Dox) in cell culture medium.

## 2. Methods

### 2.1. Cell Culture

As a model for primary hMSC, we employed the telomerized hMSC line (hMSC-TERT) which was developed in our lab [21]. The hMSC-TERT was created from primary hMSC derived from the bone marrow sample obtained from a young healthy donor, through stable overexpression of human telomerase reverse transcriptase gene (hTERT). The hMSC-TERT cells express all known markers of hMSC and “stemness” characteristics in vitro and in vivo [21, 22]. For the rest of the manuscript, we will refer to the cell line as hMSC. HEK293T is a human cell line, derived from the HEK 293 cell line and expressing a mutant version of the SV40 large T antigen, and was employed to produce recombinant viruses. Cells were cultured in Minimum Essential Medium (MEM) with 10% fetal bovine serum (FBS) and penicillin-streptomycin (P/S) (1%). All cell culture reagents were purchased from Life Technologies (Taastrup, Denmark). All the remaining chemicals were purchased from Sigma-Aldrich (Copenhagen, Denmark). Cells were incubated in 5% CO_2_, at 37°C with a humidity of 95%.

### 2.2. Lentiviral Vector Production

In order to create inducible CRISPRa cell line, we employed Tet-regulable dCas9-VP64 lentiviral expression vector (pHAGE TRE dCas9-VP64-HA, Addgene, plasmid #50916) [23]; and for inducible CRISPRi cell line, Tet-regulable dCas9-KRAB lentiviral expression vector was utilized (dCas9-TRE-KRAB-HA, Addgene, plasmid #50917) [23]. We also employed two plasmids that express lentivirus envelope proteins for lentiviral packaging and production (psPAX2 and pCMV-VSV-G, Addgene, #12259 and #8454).

HEK293T packaging cells were cultured in MEM with 10% FBS with 1% P/S until 50-60% confluence. The culture medium was changed to fresh prewarmed growth medium (without P/S) 2 hours prior to transfection. We prepared transfection DNA mixture containing dCas9-effector fusion vector, psPAX2, and pCMV-VSV-G (ratio 4 : 3 : 1) in Optimum Medium (Thermo Fisher Scientific, Roskilde, Denmark) and polyethyenimine (PEI) (1 *μ*g/*μ*l in 1x PBS, pH 4.5) at a ratio of 4 : 1 of PEI : DNA. The mixture was incubated for 5-10 min at room temperature and added gradually to the cells. The cells were incubated for 6-8 hours in 3.5% CO_2_ in a 37°C cell incubator. The medium was then replaced with fresh growth medium (3% FBS, with 25 mM HEPES) and incubated for 10 hours and mixed with sodium butyrate (10 mM). The cells continue in culture for 48 hours posttransfection.

Cell culture media were pooled from HEK293-transfected cells and stored at 4°C as the 1^st^ medium. Fresh growth medium was added (with the addition of 25 mM HEPES), and the cells were incubated overnight (60-72 hours posttransfection). The 2^nd^ medium was collected and pooled with the 1^st^ medium. One *μ*g DNase I and 1 *μ*l of 1 M MgCl_2_ per ml of viral supernatant were added, and the mixture was incubated at room temperature for 30 min to digest any carry-over plasmid DNA; this was followed by incubation at 4°C for 2-4 hours. The supernatants were filtered through a 0.45 *μ*m low protein binding filter and utilized in virus purification step. We employed the virus particle purification steps as described in [24]. Briefly, viral particles were obtained by ultracentrifugation; i.e., the collected medium supernatants were centrifuged at 80,000 g for 2 hours at 4°C using a 20% sucrose cushion. The supernatant was discarded without disturbing the pellets. Fresh collected medium supernatants were added, and a second centrifugation step was performed. Finally, the viral pellet collected from 100 ml conditioned medium, was suspended in 200 *μ*l of 1x HBSS buffer.

### 2.3. Lentiviral Transduction

hMSC line was transduced with lentiviral particles according Addgene's protocol (https://www.addgene.org/protocols/generating-stable-cell-lines/). Briefly, hMSCs were seeded and cultured until 70% confluence and infected using a range of multiplicities of infection (MOIs) (5.0 to 10.0) of the lentivirus in MEM supplemented with 8 *μ*g/ml polybrene. The cells were incubated for 48 hours, and the supernatant media was discarded to remove excess virus particles. For selection, 400 *μ*g/ml G418 (Geneticin) was used for positive section of the infected hMSC and this treatment continued for 5-7 days until all control cells (un-transduced) died. The cells were then trypsinized, and selection was continued for 2-3 weeks using culture in medium containing G418 for in order to obtain stable and pure transduced cell populations.

### 2.4. Alkaline Phosphatase (ALP) gRNA Design, Synthesis, and Transfection

Human ALP gRNA for transcription regulation was designed in an online CRISPR design tool (http://crispr.mit.edu) based on ALP promoter sequence, from -200 bp to +0 bp. The selected ALP gRNA oligo (TCGTGGCACGACCGGCCCGC**GGG**) and the universal tracrRNA oligo (Alt-R® CRISPR-Cas9 tracrRNA) were synthesized at Integrated DNA Technologies (Leuven, Belgium). The gRNA-tracrRNA guide complex was mixed as a final duplex of 10 *μ*M in nuclease-free duplex buffer (Integrated DNA Technologies) and denatured by heating at 95°C for 5 min and then allowed to form the heteroduplexes by slowly cooling to 23°C. hMSC-CRISPRi or hMSC-CRISPRa was transfected with ALP crRNA-tracrRNA guide complex employing DharmaFECT™ Transfection Reagent (Dharmacon Inc./VWR International A/S, Søborg, Denmark) according to the manufacturer's instructions (http://dharmacon.gelifesciences.com/uploadedFiles/Resources/basic-dharmafect-protocol.pdf).

### 2.5. Lipofectamine 2000 Cell Transfection and Electroporation Transfection

To compare the efficiency of gRNAs, siRNA and plasmid inhibition or overexpression in hMSCs utilizing Lipofectamine® 2000 or electroporation were performed to compare with the sgRNA transfection in hMSC-CRISPRi or hMSC-CRISPRa. Alkaline phosphatase (ALP) Silencer® Select validated siRNA was purchased from Ambion (App.Bio) (#4390821); pcDNA3-Alkaline phosphatase (ALP) plasmid was purchased from PPL (Public Protein/Plasmid Library, Jiangsu, China, #BC009647). siRNA transfection was performed by Lipofectamine® 2000 (Thermo Fisher Scientific, Roskilde, Denmark) as the manufacturer's instructions suggested for siRNA transfection in cells (https://assets.thermofisher.com/TFS-assets/LSG/manuals/Lipofectamine_2000_Reag_protocol.pdf). The plasmid transfection in hMSCs was performed by electroporation by Nucleofector™ 2b Device (Lonza, BioNordika Denmark A/S). Briefly, hMSCs were harvested and suspended with Human MSC Nucleofector Solution (Lonza, #VAPE-1001) at the concentration of 5 × 10^5^ cells/100 *μ*l, mixed with 2 *μ*g plasmid DNA, and transferred into the electroporation chamber, using program U-23. This was immediately followed by the addition of 500 *μ*l of the prewarmed culture medium containing serum and supplements; cells were then transferred into the prepared 6-well plates and, after 2 hours, were changed to fresh cell culturing medium.

### 2.6. Osteoblastic Differentiation

hMSCs were cultured to reach 80-90% confluence and then incubated in osteoblastic induction medium (OIM) containing 10% FBS, 1% Pen–Strep, 10 mM *β*-glycerophosphate, 50 *μ*g/ml 2-phosphate ascorbate, 10 nM dexamethasone, and 10 nM 1,25-dihydroxyvitamin D3. OIM medium was replaced every 3 days.

### 2.7. Adipogenic Differentiation

hMSCs were cultured to reach 95-100% confluence prior to adding adipogenic induction medium (AIM) containing MEM medium supplemented with 10% FBS, 10% horse serum, 1% Pen–Strep, 100 nM dexamethasone, 0.45 mM isobutyl methyl xanthine, 3 *μ*g/ml insulin, and 1 *μ*M rosiglitazone (Cayman, #BRL49653). The AIM medium was replaced every 2 days.

### 2.8. Chondrogenic Differentiation

For chondrogenesis in hMSCs, 250,000 MSCs were centrifuged at 500 g, 7 min in 15 ml tubes to form pellets at high-density culture. Chondrogenesis was induced for 18 days with MEM medium supplemented with 50 *μ*g/ml L-ascorbic acid-2-phosphate (Sigma-Aldrich.), 1% ITS+1 (BD Bioscience), 10^−7^ M dexamethasone (Sigma-Aldrich), and 10 ng/ml TGFb3 (R&D Systems, Wiesbaden, Germany). The aggregated cells were cultured in tubes with 0.5-1 ml medium/pellet at 37°C in a humidified atmosphere containing 95% air and 5% CO_2_. The medium was replaced every other day for 18 days [25].

### 2.9. Alkaline Phosphatase (ALP) Activity Assay

Cell viability was determined on day 7 of osteoblastic differentiation by Cell Titer-Blue cell viability assay according to the manufacturer's instructions (Promega, Nacka, Sweden). Staining intensity was measured at 579/584 by a FLUO star Omega plate reader (BMG Laboratories, Germany). ALP activity was determined by incubating the cells with 1 mg/ml of p-nitro phenyl phosphate in 50 mM NaHCO_3_ and 1 mM MgCl_2_ buffer (pH 9.6) at 37°C for 20 min. The activity was stopped by the addition of 3 M NaOH. The reaction absorbance was measured at 405 nm by a FLUO star Omega plate reader, and ALP activity was corrected for variation in cell number.

### 2.10. Alkaline Phosphatase Staining

Alkaline phosphatase (ALP) staining was performed at day 7 postinduction. The cells were rinsed with PBS and fixed in acetone/citrate (1.5 : 1, vol : vol) buffer (pH 4.2) for 5 min at room temperature. The cells were incubated for 1 hour at room temperature with ALP staining substrate solution containing 0.2 mg/ml naphthol AS-TR phosphate dissolved in distilled water (1 : 5) and 0.417 mg/ml Fast Red dissolved in 0.1 M Tris buffer.

### 2.11. Oil Red O Staining

Mature adipocyte formation was visualized on day 12 of adipocytic differentiation by staining lipid droplets with Oil Red O. Cells were washed with phosphate-buffered saline (PBS), fixed with 4% paraformaldehyde for 10 min, and then incubated with fresh-made and filtered (0.45 *μ*M) Oil Red O in 60% isopropanol solution for 1 hour at room temperature. Images were acquired using an inverted Zeiss microscope.

### 2.12. Alcian Blue Staining

To evaluate the synthesis of cartilage-specific proteoglycans, sulfated glycosaminoglycans (GAGs) were stained with Alcian blue. Cell pellets from day 18 chondrogenic differentiation were fixed and embedded by paraffin; samples were deparaffinized and hydrated to distilled water, stained in 1% Alcian blue 8-GX (Sigma-Aldrich) in 3% acetic acid in pH 2.5, and then rinsed in distilled water as previously described [26]. The accumulation of GAGs was assessed using microscopic examination.

### 2.13. Protein Sample Preparation and Western Blot Analysis

For Western blot analysis, we used whole cell lysates. The cells were washed in PBS and lysed in RIPA buffer (Thermo Fisher Scientific) supplemented with a protease inhibitor (Roche, Switzerland). Samples were centrifuged for 10 min at 13,000 rpm (4°C). Protein concentration was determined with a BCA kit (Thermo Fisher Scientific), and equal amounts of proteins were loaded on a polyacrylamide gel (Thermo Fisher Scientific). Blotted nitrocellulose membranes were incubated overnight with HA-tag primary antibody (Santa Cruz Biotechnology). The blots were developed after 1-hour incubation with secondary anti-rabbit horseradish peroxidase-conjugated antibody (Santa Cruz Biotechnology) using an ECL Western blotting kit (Thermo Fisher Scientific) and Kodak films.

### 2.14. Quantitative Real-Time PCR (qRT-PCR)

RNA from cells was isolated at day 2 of osteoblastic differentiation by TRIzol® according to the manufacturer's instructions (Thermo Fisher Scientific). The first strand complementary DNA was synthesized from 1 *μ*g total RNA by Revert aid cDNA kit (Sigma-Aldrich). RT-qPCR was performed by ABI StepOne™ Real time PCR machine with SYBR green (Thermo Fisher Scientific). The data was normalized to geometric means of reference genes and analyzed by a comparative CT method where *Δ*-CT is the difference between the CT values of the target and geometric mean of reference genes. PCR Primers for human ALP gene are as follows: ACGTGGCTAAGAATGTCATC (forward) and ACGTGGCTAAGAATGTCATC (reverse); and primers for GAPDH are as follows: GGCGATGCTGGCGCTGAGTAC (forward) and TGGTTCACACCCATGACGA (reverse).

### 2.15. Statistical Analysis

Data were collected from at least 3 independent experiments with each experiment comprising duplicates or triplicates. A one-way analysis of variance (AVOVA) test with the nonparametric Kruskal-Wallis test was used to assess statistical differences in the groups by GraphPad Prism 7.0. Data was expressed as the mean and standard deviation (SD), and *P* < 0.05 was considered as significant.

## 3. Results

### 3.1. Generation of Inducible CRISPRi or CRISPRa hMSC

**Figure 1:**
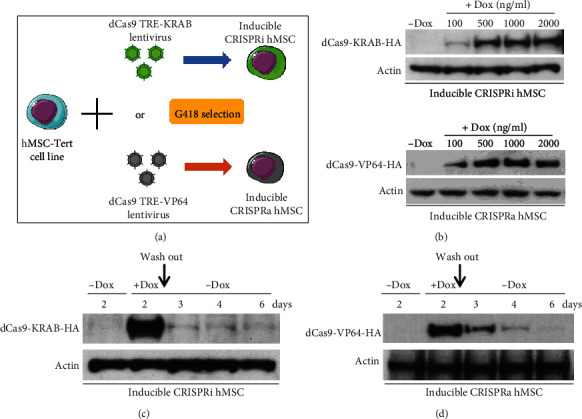
Generation of inducible CRISPRi and CRISPRa hMSCs. (a) Illustration and flow chart for generating inducible CRISPRi using dCAs9 TRE-KRAB and CRISPRa using dCas9 TRE-VP64. (b) Western blot analysis of expression of dCas9-KRAB-HA and dCas9-VP64-HA in the presence of increasing concentrations of doxycycline (Dox) in CRISPRi-hMSC and CRISPRa-hMSC, separately. The effect of the absence or presence of Dox on protein expression of dCas9-KRAB-HA (c) or dCas9-VP64-HA (d). CRISPRi-hMSC and CRISPRa-hMSC were cultured in cell culturing medium with or without Dox (0 or 1000 ng/ml) for 2 days, then washed twice by PBS and changed the cell culturing medium to the medium without Dox, with continuous culturing of the cells till day 6. Cell proteins were harvested on days 2, 3, 4, and 6 and subjected to Western blots to test the expression of dCas9-KRAB and dCas9-VP64 by HA antibody.

As shown in Figure 1(a), hMSCs were transduced with lentivirus expressing dCas9-KRAB (CRISPRi) or dCas9-VP64 (CRISPRa), which express the CRISPR-dCas9 fused with gene transcription repressor (KRAB) or activator (VP64), respectively, and the expression is driven from a doxycycline- (Dox-) inducible promoter (TRE promoter) [23]. After lentiviral transduction, cells were selected with G418 for 21-28 days in order to obtain stable expression cells (Figure 1(a)). In the absence of Dox, no expression of dCas9-KRAB or dCas-VP64 was detectable in inducible CRISPRi hMSC or CRISPRa hMSC, but when increasing concentrations of Dox adding into the culture medium, there were rapid and dose-dependent increases in their expression reaching a peak at a concentration of 1000 ng/ml (Figure 1(b)). To further prove the dynamic control of dCas9 expression in both cell lines, Dox was removed from culture medium after two-day incubation which led to the disappearance of dCas9 protein expression in both hMSC-CRISPRi and hMSC-CRISPRa within 1-2 days (Figures 1(c) and 1(d)).

### 3.2. Characterization of Inducible CRISPRi and CRISPRa hMSCs

**Figure 2:**
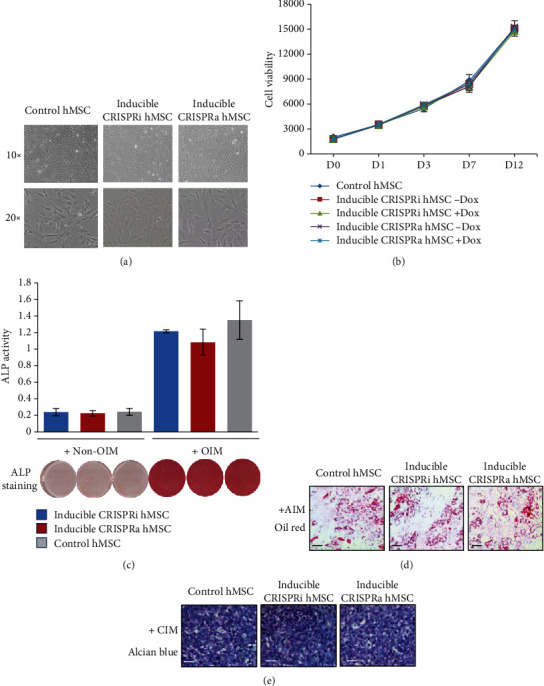
Characterization of inducible CRISPRi and CRISPRa human skeletal stem cells (hMSC). (a) The morphology of control hMSC, inducible hMSC-CRISPRi, or hMSC-CRISPRa. (b) Short-term growth curve of control hMSC, hMSC-CRISPRi, or hMSC-CRISPRa. The cells were seeded in a 96-well plate and cultured in the absence or presence of doxycycline; cell viability assay was performed at different time points during 12 days in culture (D0–D12, D = day). (c–e) Control hMSC, hMSC-CRISPRi, and hMSC-CRISPRa were induced to osteogenic, adipogenic, and chondrogenic differentiation. The cells were cultured for 7 days in osteoblast induction medium (OIM) (c), 12 days in adipocyte induction medium (AIM) (d), or 18 days in chondrogenic induction medium (CIM) (e) as described in Methods. Alkaline phosphatase (ALP) activity and ALP staining (c), Oil Red O staining (d), or Alcian blue staining (e) were performed to visualize different hMSC lineage-differentiated phenotype. Data are expressed as the means ± SD. Scale bar: 100 *μ*m.

In the presence of Dox, lentivirus-transduced hMSC-CRISPRi and hMSC-CRISPRa retained spindle-shaped fibroblast-like morphology of the parental hMSC line (Figure 2(a)). We observed no difference in cell proliferation rate between the cell lines in the presence or absence of Dox in 12 days culturing, as evidenced by determination of cell number and cell viability (Figure 2(b)). Following osteoblast (OB) differentiation induction, hMSC-CRISPRi and hMSC-CRISPRa maintained osteoblast differentiation capacity as evidenced by positive staining for ALP and induction of ALP activity (Figure 2(c)). Similarly, both cell lines differentiated readily to adipocytes or chondrocytes as compared to the parental cell line (Figures 2(d) and 2(e)).

### 3.3. Dynamic Inducible Control of ALP Transcription in hMSC-CRISPRi or hMSC-CRISPRa

**Figure 3:**
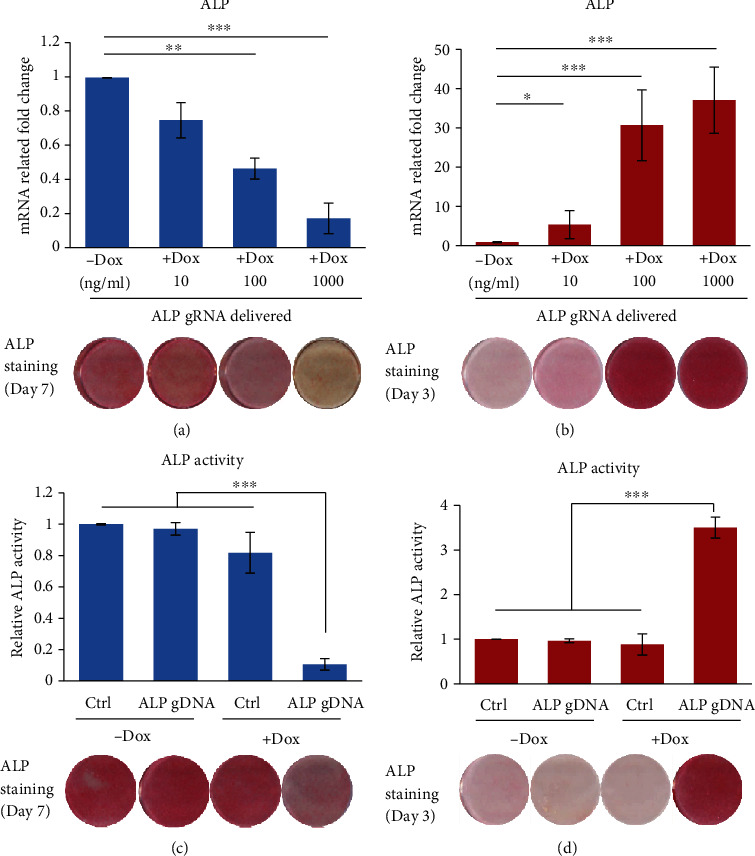
Dynamic regulation of ALP expression during osteoblast differentiation in inducible CRISPRi or CRISPRa hMSC. Inducible CRISPRi (a) or CRISPRa (b) hMSC were transfected with ALP gRNA oligo by DharmaFECT™ Transfection Reagent and cultured with increasing concentrations of Dox. ALP expression was measured by RT-qPCR at day 2. Inducible CRISPRi (c) or CRISPRa (d) hMSC was transfected with ALP gRNA oligo or negative control (Ctrl) and induced to OB differentiation in the absence or presence of Dox (1000 ng/ml). ALP activity and staining (bottom photomicrographs) were performed on day 7 in hMSC-CRISPRi or day 3 in hMSC-CRISPRa to show the most evident change for inhibition or activation. Data are expressed as the means ± SD. ^∗^*P* < 0.05, ^∗∗^*P* < 0.01, and ^∗∗∗^*P* < 0.001.

To test for regulating gene expression of specific genes in hMSC-CRISPRi or hMSC-CRISPRa, we chose alkaline phosphatase (ALP) gene as a candidate for its known role in osteoblast (OB) differentiation of hMSC [27]. One ALP gRNA was designed, and heteroduplexes were produced and transfected into hMSC-CRISPRi or hMSC-CRISPRa, respectively. After delivery in hMSC-CRISPRi, the expression level of ALP showed no change in the absence of Dox; in the presence of increasing concentrations of Dox, the gene expression of ALP exhibited dose-dependent inhibition; this was also confirmed by ALP staining (Figure 3(a)). Conversely, the expression levels of ALP gene and ALP staining were significantly increased in hMSC-CRISPRa in the presence of Dox in a dose-dependent fashion (Figure 3(b)).

### 3.4. Regulation of Osteoblast Differentiation of hMSC by Changes in ALP Gene Expression

To further validate the functional relevance of gene regulation in hMSC-CRISPRi or hMSC-CRISPRa, we compared the osteoblast differentiation capacity in hMSC-CRISPRi or hMSC-CRISPRa following transfection with ALP gRNA or negative gRNA (control (Ctrl)). We observed that in the absence of Dox, there were no differences at ALP activities in control gRNA and ALP gRNA-transfected hMSC-CRISPRi or hMSC-CRISPRa (Figures 3(c) and 3(d)), while in presence of Dox, the activity of ALP was significantly repressed in hMSC-CRISPRi or increased in hMSC-CRISPRa, and this was associated with decreased (Figures 3(c)) or increased (Figure 3(d)) ALP staining.

### 3.5. High Efficiency of Gene Inhibition or Activation in Inducible CRISPRi and CRISPRa hMSCs

**Figure 4:**
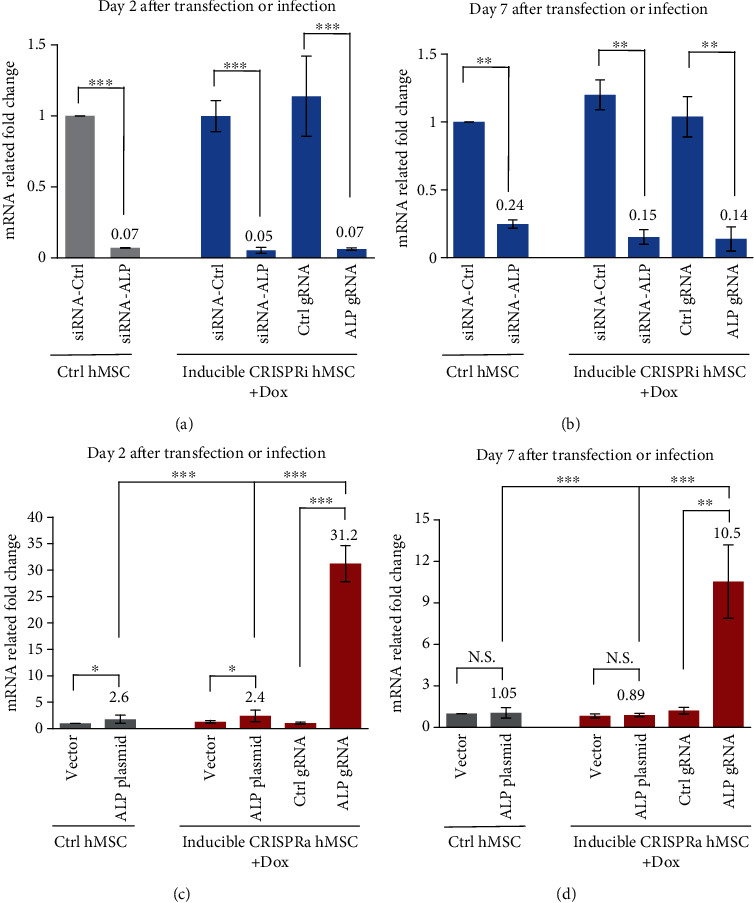
Comparison of the efficiency of gene inhibition or gene activation in control hMSC, inducible CRISPRi hMSC, and CRISPRa hMSC. (a, b) Control (Ctrl) hMSC and inducible CRISPRi were transfected with siRNA-Ctrl, siRNA-ALP, Ctrl gRNA, or ALP gRNA oligo and for 2 or 7 days as described in Methods. ALP expression was measured by RT-qPCR at day 2 (a) or day 7 (b). (c, d) Control (Ctrl) hMSC and inducible CRISPRa were transfected with pcDNA3 vector plasmid, pcDNA3-ALP plasmid by electroporation, or Ctrl gRNA and ALP gRNA oligo by DharmaFECT™ Transfection Reagent as described in Methods. ALP expression was measured by RT-qPCR at day 2 (c) and day 7 (d). Data are expressed as the means ± SD. ^∗^*P* < 0.05, ^∗∗^*P* < 0.01, and ^∗∗∗^*P* < 0.001.

To determine the efficiency of specific gene inhibition or activation by gRNA in hMSC-CRISPRi and hMSC-CRISPRa, we compared the gene inhibition with traditional siRNA transfection by Lipofectamine 2000 and the gene overexpression with traditional plasmid transfection by electroporation that we had tested before as the most efficient transient transfections in hMSCs. As shown in Figure 4, both specific siRNA and gRNA transfections in CRISPRi hMSC have significant gene inhibition on day 2 (>90%) or day 7 (>75-85%) after cell transfection (Figures 4(a) and 4(b)). On the other hand, the gene activation by gRNA in CRISPRa hMSC was shown to be much stronger than traditional plasmid overexpression (31-fold vs 2.5-fold on day 2 after transfection) (Figure 4(c)), and the gene activation by gRNA in CRISPRa hMSCs also lasted much longer: 7 days posttransfection, cells demonstrated 10 times overexpression while the plasmid-transfected overexpression effects disappeared (Figure 4(d)).

## 4. Discussion

Human bone marrow stromal/stem cells (hMSC) are multipotent cells with the ability to differentiate into osteogenic, chondrogenic, and adipogenic lineages. The cells have been utilized in molecular studies aimed at understanding the molecular mechanisms controlling lineage fate determination through targeting specific genes or genetic pathways [3, 5, 28]. Manipulating gene expression in hMSC by plasmid transfection is the most common approach. However, this approach requires specific expensive electroporation instruments or employing relatively toxic chemical formulations, and it usually exhibits low efficiency (usually <25% in our laboratory). Conversely, viral gene delivery is the most efficient way to attain stable gene expression in hMSC; however, this method requires specific laboratory setup, with time-consuming and technical difficulties.

**Figure 5:**
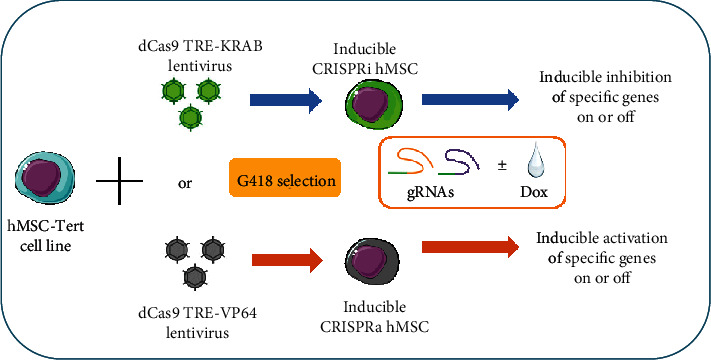
The technical flow chart of inducible inhibition or activation-specific genes in CRISPRi or CRISPRa human stromal/stem cells (hMSCs). Human bone marrow stromal/stem cell line (hMSC-TERT) stable transfected with dCas9 TRE-KRAB or dCas9TRE-VP64 to establish the inducible CRISPRi or CRISPRa cell lines. In these cells, with simply transfecting the small molecular guide RNAs (gRNAs) that target different specific genes, it can easily inhibit or activate specific gene expressions with or without doxycycline (Dox) in cell culturing mediums or differentiation induction medium.

In the present study, we employed CRISPR-dCas9 technology and created two universal hMSC lines to be utilized in specific gene transcriptional inhibition or transcriptional activation. We demonstrate that this technology did not affect the growth rate or the functional characteristics of the cells. Moreover, we demonstrated that regulation of gene expression can be achieved by the presence Dox in culture medium that acts as a “switch” to regulate gene expression. To regulate gene expression, a simple transfection by one sgRNA for targeting gene was enough to obtain either inhibition of target gene in hMSC-CRISPRi cells or overexpression in hMSC-CRISPRa cells (summary as shown in Figure 5).

We employed one transcriptional repressor, KRAB domain of Kox1, an efficient repressor of gene transcription [20], to construct with dCas9 and make the hMSC-CRISPRi cells, and we obtained 60-99% inhibition efficiency using only one sgRNA. To further enhance the inhibition efficiency, previous studies have suggested a number of other approaches, including screening of several sgRNAs to identify the most efficient sgRNA [20]; the pooling of several designed sgRNAs of the targeting gene [16, 29]; or combining several fusion transcriptional repressors with dCas9 in the system, such as KRAB, the CS (chromoshadow) domain of HP1a, the WPRW domain of Hes1, and four concatenated copies of the mSin3 domain (SID4X) [14, 16]. We observed that transfection of sgRNAs in CRISPRi cell system resulted in similar levels of gene expression inhibition compared to siRNA-mediated gene inhibition. This is expected as both are small RNA molecules with high transfection efficiency (usually >95% transfection efficiency got in hMSCs for small RNA transfection in our laboratory). However, sgRNA-CRISPRi has the important advantage of the ability to control gene expression by Dox, allowing gene manipulation at different time points at developmental stages of differentiating hMSC.

We observed high efficiency for gene regulation by CRISPR-dCas9 in CRISPRa our activation system. By simply transfecting one small molecular sgRNA of the targeting gene, we routinely obtained 5 to 20-fold overexpression for the targeting genes in hMSCs. This method is much easier than the traditional approaches for gene overexpression, e.g., transfection or infection of gene open reading frame (ORF) cloning plasmid or viral vectors, respectively. We have utilized the VP64 activator in the CRISPRa cell system. Other activator fusion proteins have the employed activation of gene expression such as P65 activator, heat shock factor 1 (HSF1), and the viral replication and transcription activator (RTA) [30]. Among these, VP64 infection was proven to demonstrate more efficiency than other reported activities [11]. In addition, several approaches have been described to further enhance gene activation in CRISPRa system, e.g., fusing multirepeats of one transcriptional activator with dCas9 [20, 31]; combining several different transcriptional activators together with dCas9 [32]; or using multiple sgRNAs designed across the targeting gene promoter [19].

One of the most striking advantages for CRISPRi and CRISPRa is the possibility for simultaneous multigene targeting [19, 20, 29]. Through single transfection with several sgRNAs targeting different genes, the method can inhibit or enhance multiple genes allowing examination of the combined effects of multigene inhibition or activation. Moreover, VP64-CRSIPRa and KRAB-CRISPRi hMSC lines can also be employed in screening a large number of effectors by sgRNAs libraries. Combing both CRISPRi and CRISPRa together to study one or several targeting genes by loss-or-gain effects can further help confirming the specific effects of targeting factors and limit the bias of function study.

CRISPR/Cas9 technology is a powerful tool for creating gene knock-ins and knock-outs; however, concerns need to be addressed consequentially to mutations engendered at gene sites other than the intended target site (off-target). The selection and design of the sgRNA for specific target genes are the key to control the specificity of targeting in our system. We suggest initially selecting several highest scored sgRNAs for the target gene that are designed by different programs. Alternatively, commercially proved sgRNAs (Thermo Fisher Scientific, Merck/Sigma, Takara et al.) are now available. Pooling of several sgRNAs for one target gene might improve the inhibition or activation efficiency of the target gene; however, limiting the number of sgRNAs can benefit the reduction of off-target effects. If one sgRNA has a high enough efficiency, we suggest using one sgRNA targeting for one gene. Several different sgRNAs for one gene can be used in different parallel experiments to obtain consistent results and limit misleading the effects of off-targeted events. Moreover, besides checking the specific target gene, testing the several highest potential off-target genes by the gRNAs can also help to confirm the targeting specificity in the CRISPR cells.

## 5. Conclusion

The availability of inducible hMSC-CRISPRi and hMSC-CRISPRa cell lines makes it possible to investigate the role of specific genes and genetic pathways at a specific developmental stage of hMSC differentiation and map the genetic regulatory networks underlying lineage differentiation of hMSC. These tools can help to enhance our understanding of hMSC biology and are also relevant to regenerative medicine applications for tissue regeneration.

## Data Availability

The data used to support the findings of this study are included within the article. The materials used to support the findings of this study are available from the corresponding author upon request.

## Abbreviations

hMSCs: Human bone marrow stromal/stem cells
Dox: Doxycycline
ALP: Alkaline phosphatase
Type II CRISPR-Cas9 system: Clustered Regularly Interspaced Palindromic Repeats–CRISPR-associated 9
sgRNA: Single guide RNA
DSB: Double-stranded break
KRAB: Krüppel-associated box
hMSC-TERT: Telomerized hMSC line
hTERT: Human telomerase reverse transcriptase gene
MEM: Minimum Essential Medium
FBS: Fetal bovine serum
P/S: Penicillin-streptomycin
dCas9-TRE-KRAB: Tet-regulable dCas9-KRAB lentiviral expression vector
pHAGE TRE dCas9-VP64: Tet-regulable dCas9-VP64 lentiviral expression vector
PEI: Polyethyenimine
G418: Geneticin
OIM: Osteoblastic induction medium
AIM: Adipogenic induction medium
PBS: Phosphate-buffered saline
qRT-PCR: Quantitative real-time PCR
OB: Osteoblast
Ctrl: Control
CS: Chromoshadow
ORF: Open reading frame.

## Additional Points

*Highlight*. (i) Using the principle of CRISPR technology, we established two human stromal/stem cell lines: CRISPRi and CRIPSRa. (ii) These two cells overcome the difficulty of plasmid transfection in hMSCs and make gene transcription inhibition and overexpression easily by only transfecting a small guide RNA (gRNA). (iii) The inhibition and activation of specific genes have been proved to be highly efficient in both cell lines. (iv) The transcription regulation in both cell lines can be controlled by Dox induction at different times during differentiation.
